# Quantitatively analyzing the college student employment policy in China based on PMC-index model

**DOI:** 10.1371/journal.pone.0310479

**Published:** 2024-10-24

**Authors:** Changming Cui, Kangyu Wang

**Affiliations:** 1 Student Work Department, Jilin Agricultural University, Changchun, Jilin, China; 2 College of Life Science, Jilin Agricultural University, Changchun, Jilin, China; Universidad de Granada, SPAIN

## Abstract

The employment policies targeted at university graduates, which are exceedingly valuable to the national human resource, are closely related to the national economy and people’s livelihood. The employment policies for college students are essential pathways for promoting high-quality job placement for graduates, making the review and evaluation of relevant policies exceedingly significant. This study analyzes the university-student employment policies issued between 1997 and 2023; the researchers utilize text mining and content analysis and refer to the policy indicators designed by existing scholars to develop an evaluation index for college student employment. A PMC index model was constructed to quantitatively evaluate 9 selected policy samples. The results indicate that P5, P6, and P7 have good consistency ratings; P8, P4, and P9 have acceptable consistency ratings; and P2, P3, and P1 have low consistency ratings. The average score for the 9 policy samples is 5.03, reflecting an overall satisfactory quality of China’s college student employment policies. By constructing a PMC surface, the model visually reveals certain deficiencies in China’s college-student employment policies in regard to policy design, policy tools, and policy content. This study provides countermeasures and proposals from aspects such as policy nature and policy measures.

## 1. Introduction

Education for the party and country is the fundamental task of higher education, and employment is a crucial symbol for comprehensively testing the effect of higher education. The report of the 20th National Congress of the Communist Party of China clearly highlighted that talent is the first resource: employment is the most basic form of livelihood. The report also proposes the following measures: implement the employment priority strategy, strengthen the employment priority policy, enhance the employment promotion mechanism, and promote high-quality full employment [1]. The Central Economic Work Conference proposes that the implementation of the employment policy should be more prominent in promoting the employment of youth, especially college graduates. Premier Li Qiang has made several deployments at the national "Two sessions" and The State Council’s Executive Meeting. Currently, there remains a certain gap in high-quality employment in colleges and universities nationwide, and for college graduates, the promotion of high-quality employment cannot be separated from the government’s guidance and policy support. Recently, the state has issued a series of policy documents to promote smooth employment and high-quality employment for college graduates, such as the Notice on the issuance of the 14th Five-Year Plan for Employment Promotion and the Notice on Further Improving the employment and Entrepreneurship of college graduates and other youth. These policy documents have critically impacted the development process of high-quality employment for college students.

Policy evaluation is a complex project to measure the quality of policies according to specific evaluation procedures and standards [2]. Scholars’ research on policy evaluation mainly focuses on the methods of policy evaluation, that is, using different evaluation methods to evaluate policies in different types of fields. For example, Ma et al. evaluated and studied intellectual property protection policies based on entropy weight TOPSIS (approximate ideal solution sorting) method [3]. Wang et al. applied Delphi method to construct the evaluation index system of health integration into all policies in earthquake-stricken areas [4]. The accuracy and effectiveness of policy evaluation results depend on the scientificity and objectivity of policy evaluation methods. Therefore, the research and application of policy evaluation methods become particularly important.

Currently, in foreign countries, some countries have actively formulated and promulgated relevant policies and regulations. In 2002, Germany formulated and promulgated laws and regulations such as the "Part-time Work Law" and "Employment Promotion Law", which provide corresponding legal support for college students’ employment and entrepreneurship. Japan defines the employment rights of college students and the obligations of the government and society from the policy-legalization perspective. In the labor field that includes the employment of college graduates, a somewhat complete labor and employment legal system is formed by the Constitution of Japan, the Labor Standards Law and so on. Domestic research on the employment policy of college graduates mainly focuses on the development and reform of college graduates’ employment policy, policy evaluation and optimization. The relevant researches mainly focus on the external environment and implementation effect of the policy, and pay less attention to the content of the policy text itself. There are few literatures on the evaluation and optimization of employment policies of college graduates based on the policy text and the characteristics of employment policies of college graduates. In terms of policy development and reform, different scholars divide the employment policy of college graduates into different stages according to the economic and social development conditions in different periods of our country[5]. For example, Li et al. divided the employment policy of college graduates in China into three development periods: state deployment, unified recruitment (1949–1993), two-way choice, independent employment (1994–2007), and mass entrepreneurship and high-quality employment (2008—present) [6]. Zhang pointed out that since the 1990s, the employment policy of college graduates has gone through five stages: The transition phase of smooth transition and gradual adjustment (1993–2001), the phase of promoting comprehensive reform in response to challenges (2002–2007), the phase of responding to crises and implementing more active employment policies (2008–2012), and the phase of integrated innovation in response to transformation, encouraging entrepreneurship and promoting employment (2013–2018). Advance with The Times, deal with domestic and foreign complex factors of employment priority strategy promotion phase (2019 to date) [7]. In terms of policy evaluation and optimization, Yang discusses the optimization and upgrading of college students’ employment policy from three aspects: realistic logic, realistic dilemma and promotion strategy [8]. Shen optimized the graduate employment policy by constructing a two-dimensional analysis framework of "policy tools" and "employment channels" [9]. The research on the textual analysis of employment policies for university graduates in China primarily focuses on transforming employment policies for university graduates, assessing policy content, the determinants affecting these policies, and recommendations of policy [10–16].

Currently, most scholars focus on the effectiveness of policy implementation, the selection and utilization of policy tools, the level and evaluation of domestic policies in a specific location, and the inspiration and comparison of relevant policies in developed countries; moreover, they apply methods including text analysis to explore and analyze such topics [17–19]. The effectiveness of policy implementation is critically dependent on policy sample consistency. There is a dearth of research on the outcomes of China’s higher education employment policies. Therefore, this study aims to examine the consistency of the employment policies for college students in China, determine whether these policies have achieved their intended effects, and explore whether further optimization can be achieved. These questions necessitate more detailed and in-depth research. This study innovatively uses the PMC model to quantify the effect of employment policies in Chinese universities, combines policy text mining with the construction of PMC-Index model for the first time, and uses the method of text mining to obtain the internal raw data of policy texts on the basis of content analysis, quantitative analysis and qualitative analysis, so as to conduct a quantitative evaluation of China’s higher education employment policies. To a certain extent, it ensures the objectivity of policy evaluation, improves the accuracy of policy evaluation, and then provides a reference for the optimization of China’s higher education employment policy.

## 2. Data and methods

### 2.1 Data sources

To comprehensively collect the policy texts, the following retrieval strategies are mainly adopted: (1) search for keywords such as "university employment" and "university student employment" in literature databases such as CNKI, search relevant literature, and possess a certain understanding of the relevant research on university employment policies. (2) Search relevant policy texts on the official websites of national governments and relevant departments. (3) Search for keywords such as "university employment" and "college student employment" through authoritative and professional data websites such as "Peking University Magic Weapon" to query relevant policy texts. (4) Conduct a keyword search through search engines such as Baidu to compensate for missing relevant policy documents. Through the preliminary collection of policy texts, 58 university employment policy texts issued at the national level were finally determined (the search time was up to December 2023).

Second, the collected policy texts are screened by manual screening. After selecting policy texts related to university employment policies, policy texts exhibiting limited practical significance such as cases and news on university employment were deleted. In addition, informal policy texts such as solicitation drafts, letters, and approval responses were excluded; thus, only policy texts with high authority could be selected. After screening and sorting out, 9 policies issued by relevant departments at the national level were finally selected as research objects (Table 1).

**Table 1:** Sample employment policies of Chinese universities.

Serial number	Policy name	Release time	Publication size	Issuing authority
P1	Interim provisions on the employment of graduates of ordinary institutions of higher learning	March, 1997	Teaching (1997) No. 6	State Education Commission
P2	Some opinions on strengthening the training of service outsourcing talents and promoting the employment of college graduates	March, 2009	Jiaogao (2009) No. 5	Ministry of Education, Ministry of Commerce
P3	Opinions implementing measures aimed at strengthening employment stability in response to the impact of the COVID-19 epidemic	March, 2020	Guo Banfa (2020) No. 6	State Council
P4	Notice on the issuance of the "14th Five-Year Plan" Employment Promotion Plan	August, 2021	Guofa (2021) No. 14	State Council
P5	Notice on Further Implementing the Demonstration Action to Promote Entrepreneurship and the Employment of college Graduates	February, 2022	Development of High Technology (2022) No. 187	National Development and Reform Commission, Ministry of Education, Ministry of Industry and Information Technology, Ministry of Human Resources and Social Security, Ministry of Agriculture and Rural Affairs, State-owned Assets Supervision and Administration Commission of the State Council, Central Committee of the Communist Youth League, and All-China Women’s Federation
P6	Notice on implementing the plan for developing millions of job apprenticeship positions	March, 2022	Ministry of Human Resources and Social Security (2022) No. 11	Ministry of Human Resources and Social Security, Ministry of Education, Ministry of Science and Technology, Ministry of Industry and Information Technology, Ministry of Civil Affairs, Ministry of Finance, Ministry of Commerce, State Council, Central Committee of the Communist Youth League, and All-China Federation of Industry and Commerce
P7	Notice on Further Improving the Employment and Entrepreneurship of college graduates and other youth	May, 2022	Guo Banfa (2022) No. 13	State Council
P8	Notice on Promoting social organizations to further assist college graduates and other groups in employment	July, 2022	Minfa (2022) No. 57	Ministry of Civil Affairs, Ministry of Education, Ministry of Human Resources and Social Security
P9	Circular on Optimizing and Adjusting Policies and Measures for stabilizing Employment to Promote Development and benefit People’s Lives	April, 2023	Guo Banfa (2023) No. 11	State Council

### 2.2 Research methodology

The Policy Modeling Consistency index model is a commonly utilized quantitative evaluation policy method that is mainly utilized for evaluating policy consistency. The index incorporates diversified disciplinary theories into its development system and adopts a model combining quantitative and qualitative models; Thus, it scientifically evaluates the significance of policy issuance and the policy-implementation impact [20]. Using the PMC-index model, researchers can evaluate the internal consistency of policies, and the degree of concave surface is regarded as a standard for evaluating the quality of policies through the curved surface map. It should be noted that there is no explicit requirement on the quantity of variable parameters for constructing the PMC-index model, and the weights of each variable are similar. Therefore, the model exhibits the following advantages: it can equally and comprehensively consider the influence of variable factors, and clearly and intuitively reflect the policy-evaluation results through the curved surface diagram. To establish a relatively excellent PMC-index model, four steps must be followed: variable selection and parameter identification, multi-input output table preparation, PMC index calculation for each policy, and PMC-surface drawing for each policy. Currently, the PMC model is widely utilized by scholars to quantify the policy effect research in domains including science and technology, medicine, education, industry, and economy [21–24].

#### 2.2.1 Quantity selection and parameter setting

All relevant and possible variables must be considered before establishing the employment-policy evaluation model in colleges and universities. In the selection of variables, it is necessary to carefully analyze the content of the policy text and exhaustively tap the effective in-text information. Herein, ROSTCM6 software is utilized to extract high-frequency words in the policy text; some high-frequency words without apparent significance are removed, and high-frequency words are summarized; and principal variables and sub-variables are subsequently determined. According to the research conducted by Estrada and other relevant scholars, 9 principal variables and 53 sub-variables were finally determined as the basis for policy text evaluation (Table 2).

**Table 2:** Quantitatively setting the quantitative evaluation of an employment policy in Chinese universities.

Main variable	Sub-variable	The formulation of based on reference
Policy Nature (X_1_)	Prediction (X_1,1_); Recommendations (X_1,2_); Feedback (X_1,3_); Supervision (X_1,4_); Description (X_1,5_); Orientation (X_1,6_).	Estrada [24]
Policy Issuing Agency (X_2_)	Ministry of Human Resources and Social Security (X_2,1_); Ministry of Education (X_2,2_); Ministry of Science and Technology (X_2,3_); Ministry of Industry and Information Technology (X_2,4_); Ministry of Civil Affairs (X_2,5_); Ministry of Finance (X_2,6_); Ministry of Commerce (X_2,7_); State Council (X_2,8_); Communist Youth League Central Committee (X_2,9_); All-China Federation of Industry and Commerce (X_2,10_).	Estrada [24]
Policy Audience (X_3_)	Local government (X_3,1_); Enterprise(X_3,2_); Universities (X_3,3_); Social organization (X_3,4_).	Gao et al. [12]
Policy Measure (X_4_)	Objective planning (X_4,1_); Financial support (X_4,2_); Publicity report (X_4,3_); Public services (X_4,4_); Talent support (X_4,5_); Infrastructure (X_4,6_); Technology support (X_4,7_); Demonstration Pilot (X_4,8_).	Gao et al. [12]
Policy Prescription (X_5_)	Long term (X_5,1_); Metaphase (X_5,2_); Short term (X_5,3_).	Estrada [24]
Policy Design (X_6_)	Guiding ideology (X_6,1_); Basic principles (X_6,2_); Main objective (X_6,3_); Key tasks (X_6,4_); Organization and leadership (X_6,5_); International cooperation (X_6,6_); Publicity and education (X_6,7_); Inspection Inspector (X_6,8_).	Liu et al. [15]
Policy Instrument (X_7_)	Command control (X_7,1_); Motivational type (X_7,2_); Guide type (X_7,3_); Voluntary participation (X_7,4_).	Gao et al. [12]
Policy Content (X_8_)	Professional skills (X_8,1_); Recruitment scale (X_8,2_); Education and training (X_8,3_); Employment assistance (X_8,4_); Resource sharing (X_8,5_); Specification standards (X_8,6_); Public Service (X_8,7_).	Gao et al. [12]
Policy Evaluation (X_9_)	Well-founded (X_9,1_); Clear goals (X_9,2_); Detailed solutions (X_9,3_).	Wang et al. [11]

The setting of variable parameters: After the principal variable is determined, the selection of sub-variables should comprehensively consider the policy-related influencing factors, and the weight of each sub-variable remains constant. The sub-variable values must follow the [0,1] distribution, and the value can be 0 or 1. When the policy content involves related variables, the value is 1; If this variable is not involved, the value is set at 0. Consider X_1,1_ in Table 1 as an example. If the policy text is predictive, the policy X_1,1_ is assigned to 1; otherwise, the policy X_1,1_ is assigned to 0.

#### 2.2.2 Construction of multi-input output table

This section establishes an alternative data analysis framework that can store large amounts of data; thus, each variable can be calculated. Using the established multi-input-output table, 9 principal variables and their sub-variables are calculated. There is no limit on the number of sub-variables, and the importance of each sub-variable is the same, without the need for importance ranking (Table 3).

**Table 3:** Multi-input-output table of the quantitative evaluation of employment policies in Chinese universities.

Main variable	X_1_	X_2_	X_3_	X_4_	X_5_	X_6_	X_7_	X_8_	X_9_
Sub-variable	X_1, 1_ X_1, 2_ X_1, 3_ X_1, 4_ X_1, 5_ X_1, 6_	X_2, 1_ X_2, 2_ X_2, 3_ X_2, 4_ X_2, 5_ X_2, 6_ X_2, 7_ X_2, 8_ X_2, 9_ X_2, 10_	X_3, 1_ X_3, 2_ X_3, 3_ X_3, 4_	X_4, 1_ X_4, 2_ X_4, 3_ X_4, 4_ X_4, 5_ X_4, 6_ X_4, 7_ X_4, 8_	X_5, 1_ X_5, 2_ X_5, 3_	X_6, 1_ X_6, 2_ X_6, 3_ X_6, 4_ X_6, 5_ X_6, 6_ X_6, 7_ X_6, 8_	X_7, 1_ X_7, 2_ X_7, 3_ X_7, 4_	X_8, 1_ X_8, 2_ X_8, 3_ X_8, 4_ X_8, 5_ X_8, 6_ X_8, 7_	X_9, 1_ X_9, 2_ X_9, 3_

Note: X_1_ stands for Policy Nature, X_2_ stands for Policy Issuing Agency, X_3_ stands for Policy Audience, X_4_ stands for Policy Measure, X_5_ stands for Policy Prescription, X_6_ stands for Policy Design, X_7_ stands for Policy Instrument, X_8_ stands for Policy Content, X_9_ stands for Policy Evaluation.

#### 2.2.3 Calculating the PMC-index model

Currently, the calculation of the PMC-index model is usually divided into the following steps: First, the secondary index is assigned using Formula (1). Second, as per Formula (2), the second level indicators under the same first level indicators are summed to obtain the value of each level indicator. Finally, as per Formula (3), the PMC-index score of the corresponding policy is calculated.

X∼N[0,1] (1)

X={XR:[0∼1]} (2)

$$X_{i}[\sum_{j=1}^{n}\frac{X_{ij}}{T(X_{ij})}]$$ (3)

$$PMC-Index=\sum_{i=1}^{m}\left(X_{i}[\sum_{j=1}^{n}\frac{X_{ij}}{T(X_{ij})}]\right)$$ (4)

where i denotes first-order variables, i = 1,2,3,… m; j denotes the second-order variable, j = 1,2,… n; and T denotes the number of all secondary variables. The PMC-index indicates scores pertaining to the policy-consistency degree.

According to the PMC-index score, the policy consistency level is divided into four levels: [0,3.99] denotes low consistency, [4,5.99] denotes acceptable consistency, [6,7.99] denotes good consistency, and [8,9] denotes excellent consistency. When the score of PMC model results is larger, the policy-text content is more comprehensive; the stronger the policy consistency, the stronger the operability of the policy in the implementation process (Table 4).

**Table 4:** PMC-index policy-evaluation-standard indicators.

PMC-index score	0–3.99	4–5.99	6–7.99	8–9
Consistency level	Low (L)	Acceptable (A)	Good (G)	Excellent (E)

Note: E stands for Excellent, G stands for Good, A stands for Acceptable, L stands for Low.

#### 2.2.4 PMC-surface drawing

Based on the calculation results of the PMC-index model, three-dimensional images of PMC-surfaces can be built to graphically reflect the advantages and disadvantages of college-student employment policy samples [25]. Herein, a matrix with 3 rows and 3 columns is established as per X_1_-X_9_ and calculated according to Eq (5).


$$PMC-Surface=[\begin{array}{ccc} X_{1} & X_{4} & X_{7}\\ X_{2} & X_{5} & X_{8}\\ X_{3} & X_{6} & X_{9} \end{array}]$$ (5)

## 3.Results and analysis

### 3.1 PMC-index model results of policy samples

Based on the evaluation system of China’s college student employment policies, this study established a multi-input-output table for each variable, obtained the results of the PMC index model through calculation, and determined the pros and cons of policies as per the scores, as depicted in Table 5.

**Table 5:** The score of policy samples and its evaluation results.

Main variable	P1	P2	P3	P4	P5	P6	P7	P8	P9
X_1_	0.33	0.33	0.33	0.5	0.67	0.50	0.33	0.33	0.5
X_2_	0.1	0.2	0.1	0.1	1	0.8	0.1	0.3	0.1
X_3_	0.5	0.75	0.75	1	1	0.75	1	0.75	1
X_4_	0.25	0.38	0.38	0.5	0.88	1	1	0.88	0.5
X_5_	0.33	0.33	0.33	0.33	0.33	0.33	0.33	0.33	0.33
X_6_	0.5	0.38	0.5	0.5	0.75	0.88	0.88	1	0.38
X_7_	0.25	0.25	0.25	0.5	0.5	0.25	0.5	0.5	0.25
X_8_	0.43	0.43	0.29	0.71	1	1	1	0.86	0.29
X_9_	0.67	0.67	0.67	1	1	0.67	1	1	0.67
PMC-index	3.36	3.72	3.6	5.14	7.13	6.18	6.14	5.95	4.02
Ranking	9	7	8	5	1	2	3	4	6
Results	L	L	L	A	G	G	G	A	A

Note: G stands for Good, A stands for Acceptable, L stands for Low.

According to the results in Table 5, the result score of the PMC-model selected from 9 policy samples increased from 3.36 to 7.13, with an average value of 5.03, thus indicating an overall upward trend: 3 policy consistency grades are good, 3 policy consistency grades are acceptable, and 3 policy consistency grades are low. Because these 9 policies are selected in chronological order, the research indicates that the rationality, innovation, and integrity of university employment policies are gradually enhanced.

The PMC-index score of P5 is the highest (7.13), thereby indicating that the policy has a good consistency. The first-level variables of P6 and P7 (ranked second and third) are also higher than a certain level, thus indicating that these two policies have also comprehensively considered various variable dimensions, and both belong to a level associated with good consistency. The PMC-index score of P1 is the lowest (3.36), which is associated with the low consistency level. Meanwhile, the index scores of P3 and P2 are lower than a certain level, and these two policies also belong to the low consistency level. The scores of P8, P4, and P9 are between 4 and 6, which is an acceptable level of consistency.

### 3.2 PMC-surface results of policy samples

Based on the calculation results of the PMC-index, this section applies Formula (5) to establish a PMC-matrix corresponding to 9 policies, and the results are illustrated in Table 6.

**Table 6:** PMC-matrix of policy samples.

Policy	P1	P2	P3
PMC-matrix	$\left(\begin{array}{c} 0.33\;0.10\;0.50\\ 0.25\;0.33\;0.50\\ 0.25\;0.43\;0.67 \end{array}\right)$	$\left(\begin{array}{c} 0.33\;0.20\;0.75\\ 0.38\;0.33\;0.38\\ 0.25\;0.43\;0.67 \end{array}\right)$	$\left(\begin{array}{c} 0.33\;0.10\;0.75\\ 0.38\;0.33\;0.50\\ 0.25\;0.29\;0.67 \end{array}\right)$
Policy	P4	P5	P6
PMC-matrix	$\left(\begin{array}{c} 0.50\;0.10\;1.00\\ 0.50\;0.33\;0.50\\ 0.50\;0.71\;1.00 \end{array}\right)$	$\left(\begin{array}{c} 0.67\;1.00\;1.00\\ 0.88\;0.33\;0.75\\ 0.50\;1.00\;1.00 \end{array}\right)$	$\left(\begin{array}{c} 0.50\;0.80\;0.75\\ 1.00\;0.33\;0.88\\ 0.25\;1.00\;0.67 \end{array}\right)$
Policy	P7	P8	P9
PMC-matrix	$\left(\begin{array}{c} 0.33\;0.10\;1.00\\ 1.00\;0.33\;0.88\\ 0.50\;1.00\;1.00 \end{array}\right)$	$\left(\begin{array}{c} 0.33\;0.30\;0.75\\ 0.88\;0.33\;1.00\\ 0.50\;0.86\;1.00 \end{array}\right)$	$\left(\begin{array}{c} 0.50\;0.10\;1.00\\ 0.50\;0.33\;0.38\\ 0.25\;0.29\;0.67 \end{array}\right)$

**Fig 1:**
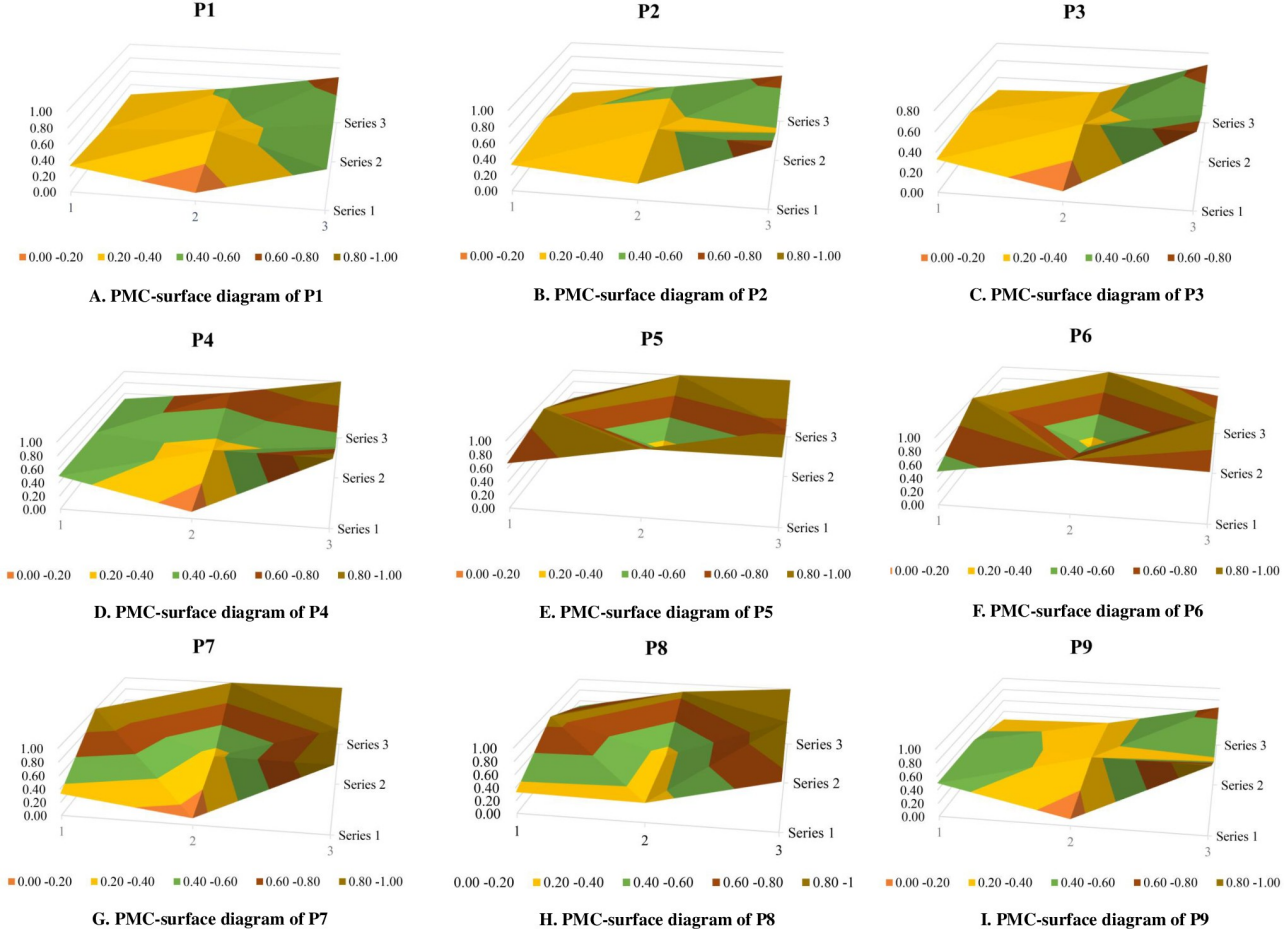
PMC-surface of digital economic policy. (A) PMC-surface diagram of P1. (B) PMC-surface diagram of P2. (C) PMC-surface diagram of P3. (D) PMC-surface diagram of P4. (E) PMC-surface diagram of P5. (F) PMC-surface diagram of P6. (G) PMC-surface diagram of P7. (H) PMC-surface diagram of P8. (I) PMC-surface diagram of P9.

In the PMC-surface diagram of 9 university employment policy samples (Fig 1A–1I), the column values are the horizontal coordinate values of the matrix, and the sequence values are the vertical values of the matrix. Different color blocks represent the scores of different variables, and the merits of each policy sample can be judged as per the image surface’s degree of depression [26].

### 3.3 Analyzing the PMC-surface results of policy samples

Combined with the calculated results of the PMC-index and the corresponding surface chart, the selected 9 university employment policies were analyzed according to the policy level and score (Fig 1A–1I). First, the P5, P6, and P7 policies exhibit good consistency. The P5 score is 7.13, ranking first. Compared with the other eight policies, P5 has significant advantages in X_2,_ X_3_, X_4_, X_6_, X_7_, X_8,_ and X_9_. "On the in-depth implementation of entrepreneurship to promote employment demonstration action to promote college graduate’s entrepreneurship and employment" is a crucial guiding document focusing on the employment of college students during the "14th Five-Year Plan" period: its policy timeliness and effectiveness level attained the highest value, and in the feedback, the policy is predicted to perform relatively well because the policy content is more comprehensive. Therefore, the rating of this policy ranks first. Moreover, this policy has exhibited considerable effectiveness in regard to aspects such as feedback and prediction, and the policy object is also relatively comprehensive. Therefore, the policy is rated excellent; P6 and P7 grades are also good.

P8, P4, and P9 consistency are acceptable. If P8 is considered as an example, the policy performs more optimally in X_6_ and X_9_; therefore, it can be inferred that the policy design and policy evaluation are relatively comprehensive, which also reflects the diversity and professionalism of the policy publication’s main body. However, in X_1_, X_2_, and X_3_ and other aspects of poor performance. This observation indicates that the predictability and supervision of the policy and the diversity of the policy audience should be strengthened, and the publicity and reporting work should be emphasized in regard to measures. Particularly, it should be noted that the P8 policy is a notice on social organizations to further facilitate the employment of groups such as college graduates, and the policy is more specific and involves fewer aspects. Therefore, the score indicator (e.g., policy audience) is relatively low.

**Fig 2:**
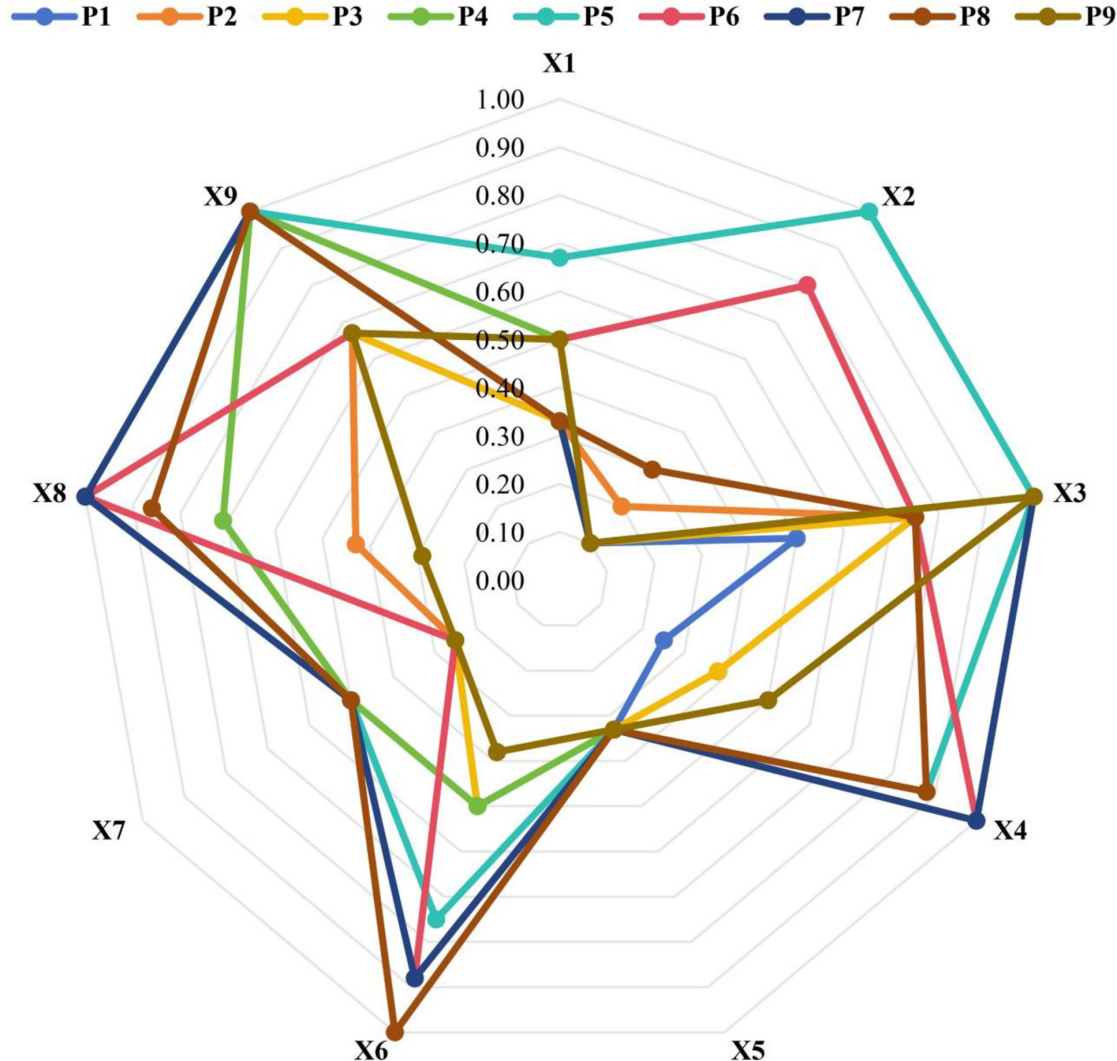
Radar map of digital economy policy.

The consistency levels of P2, P3, and P1 are lower. These three policies were released earlier, and their scores in various policy indicators were low, thereby indicating that there were many deficiencies in the early formulation of China’s university employment policies, and that the formulation of China’s employment policies gradually tended to be excellent and complete over time. The comparative analysis of policy consistency is depicted in Fig 2.

## 4. Conclusion and enlightenment

This study utilizes 58 employment-related policies of colleges and universities issued at the national level from 1997 to 2023 as the research object, compares and analyzes the consistency of policies, refers to the PMC quantitative evaluation model, and conducts quantitative evaluation and analysis on 9 selected college-employment policy texts. The research results indicate the following: (1) The 9 selected policies are classified as per their levels, among which P5, P6, and P7 have good consistency, P8, P4, and P9 are acceptable in consistency, whereas P2, P3, and P1 are low in consistency. Generally, the factors that affect policy scores mainly include policy audience, policy measures, and policy content. (2) The quality of employment policies in colleges and universities in China is generally at a good level; although relevant policy documents that are promulgated can affect a certain role, the role and effectiveness of employment policies in colleges and universities should be improved, and laws and regulations on employment policies in colleges and universities should be enhanced. (3) There is a positive correlation between the employment policy’s PMC-index score and its effectiveness level.

Based on the preceding conclusions, this study proposes the following.

First, the nature of the policy: Currently, China’s university employment-related policies mostly assume an advisory, descriptive, and guidance nature; the policies lack a forecast, feedback, and supervision nature. Some policies emphasize how to promote the high-quality development of employment in colleges and universities; however, the achievements related to the promotion of high-quality employment remain unclear. Therefore, on one hand, some policy contents with a forecasting and feedback nature should be appropriately added in the policy-formulation process. On the other hand, the government has formulated policies related to employment in colleges and universities, aiming at promoting specific goals such as the employment rate of college graduates; however, relevant contents should be appropriately increased with a focus on supervision, such as the addition of regulatory agencies.

Second, policy measures: Most of the current policies emphasize more traditional policy measures such as target planning, public services, and financial support. In regard to the rapid development of information technology and novel media, performing a good job in publicity and reporting is also a necessary policy measure. Through publicity and reporting, the masses can not only understand the practical results achieved by the current employment of college graduates in China, but also attract more enterprise forces and social forces to jointly facilitate the high-quality employment of college graduates. Meanwhile, the support for infrastructure and science and technology should continue to increase. The difference in infrastructure is not only a crucial reflection of the development gap between different regions in China, but also an urgent problem affecting the balance of education resources. To enhance the support level of science and technology, on one hand, researchers should increase the economic investment in higher education, enhance the development conditions of higher education, and build a robust foundation for educating students in colleges and universities. On the other hand, they should focus more on the ideological and moral development of students. It is necessary to promote work requirements that cultivate morality, and to further enhance the professional skills of college students and the correct attitude towards employment.

Third, university-employment policy making should focus on the current scenario affecting the unbalanced education development in China. In the report of the Party’s 20th National Congress, the three strategies of education, science and technology, and talent were novelly unified. Universities in all regions should exhaustively harness their own subjective initiative, formulate more targeted employment guidance policies as per the level of local economic development, and strengthen inter-regional exchanges and cooperation in employment-related fields. The employment problem of college graduates is becoming increasingly critical. Colleges and universities in the eastern region with a relatively high economic level have significant advantages in economy, science, and technology; they can, therefore, support western region-based colleges and universities with relatively backward development in experience and technology. Universities in the western region can exhaustively harness their advantages in aspects such as environment and geography to promote the coordinated development of employment in Chinese universities in the direction of high quality.

Policy formulation and implementation is a process of dynamic adjustment. The purpose of establishing PMC index evaluation model is to find the relatively weak link of the current policy by comparing the index system. This paper applies this model to the evaluation of employment policies of college graduates, and establishes a policy evaluation index system based on the text characteristics of employment policies of college graduates. Through vertical comparison of indicators within policies and horizontal comparison between different policies, the direction of policy improvement is clarified. Although this paper has conducted a more in-depth discussion on the quantitative evaluation of the employment policy of college graduates in China, there are still certain limitations. When constructing the evaluation system of the employment policy of college graduates in China, the setting of indicators and the evaluation dimension are greatly influenced by human subjectivity, and the variables can be optimized and upgraded in the future [27]. This paper tries to evaluate the employment policy of college graduates in China more comprehensively and accurately, and provide theoretical basis and reference for the design and formulation of other related policies [28].
